# Familial *NSD1* Exon 3 Deletion Associated with Phenotypic and Epigenetic Variability

**DOI:** 10.3390/genes16101190

**Published:** 2025-10-13

**Authors:** Sunwoo Liv Lee, Alison Foster, Dalit May, Ciara Batterton, Eguzkine Ochoa, Bryndis Yngvadottir, Ruth Armstrong, Meena Balasubramanian, Mary O’Driscoll, Marc Tischkowitz, France Docquier, Fay Rodger, Ezequiel Martin, Ana Toribio, Eamonn R. Maher

**Affiliations:** 1 Department of Genomic Medicine, University of Cambridge, Cambridge CB2 0QQ, UK; sl852@medschl.cam.ac.uk (S.L.L.); by215@medschl.cam.ac.uk (B.Y.);; 2 Clinical Genetics Unit, Birmingham Women’s and Children’s NHS Trust, Birmingham B15 2TG, UK; alison.foster10@nhs.net (A.F.);; 3 Peninsula Clinical Genetics, Royal Devon and Exeter NHS Trust, Exeter EX1 2ED, UK; 4 West Midlands Genomics Laboratory, Birmingham Women’s and Children’s NHS Trust, Birmingham B15 2TG, UK; ciara.batterton@nhs.net; 5 Department of Clinical Genetics, Cambridge University Hospitals NHS Trust, Cambridge CB2 0QQ, UK; 6 Division of Clinical Medicine, University of Sheffield, Sheffield S10 2RX, UK; 7 Sheffield Clinical Genetics Service, Sheffield Children’s NHS Foundation Trust, Sheffield S10 2TH, UK; 8 Stratified Medicine Core Laboratory NGS Hub, Cambridge Biomedical Campus, Cambridge CB2 0QQ, UK; 9 Aston Medical School, College of Health and Life Sciences, Aston University, Birmingham B7 4ET, UK

**Keywords:** sotos syndrome, atypical sotos syndrome, developmental disorder, *NSD1*, DNA methylation, episignature

## Abstract

Background: Germline pathogenic variants in *NSD1* cause Sotos syndrome, a developmental disorder characterised by overgrowth, intellectual disability, macrocephaly, developmental anomalies, and, in some cases, tumour development. Familial cases of Sotos syndrome are rare and genotype–phenotype correlations are not well described. NSD1, a lysine-specific histone methyltransferase, is an important epigenetic regulator and pathogenic variants in *NSD1* are associated with a distinctive blood DNA methylation pattern (episignature). We described a family with an *NSD1* exon 3 deletion and an atypical clinical phenotype. Methods: DNA episignature profiling was undertaken with a next generation sequencing-based approach. Results: Within the family, the three affected individuals showed clinical variability with the proband being most severely affected, although none showed unequivocal macrocephaly or the characteristic facial features of Sotos syndrome. DNA methylation profiling was performed in the three affected family members, eight individuals with Sotos syndrome, and compared to control samples. The eight individuals with Sotos syndrome displayed genome-wide hypomethylation as previously described. DNA hypomethylation was also apparent in the three family members with the *NSD1* exon 3 deletion with the proband being most similar to the episignature observed in confirmed Sotos syndrome patients. The two more mildly affected relatives had less pronounced DNA hypomethylation. Conclusions: A familial germline exon 3 *NSD1* deletion was associated with mild Sotos syndrome phenotype with variable expressivity and a DNA methylation episignature that was less marked in milder cases than in individuals with classical Sotos syndrome. These findings support the use of methylation episignature analysis to explore intrafamilial variability in chromatin disorders.

## 1. Introduction

Sotos syndrome (SS, MIM#117550) is a developmental disorder characterised by pre- and postnatal overgrowth, facial dysmorphisms, macrocephaly, developmental delay, and intellectual disability. A variety of tumours, most commonly teratoma or neuroblastoma, occur in about 3% of individuals with Sotos syndrome [1,2]. Most cases of Sotos syndrome are caused by haploinsufficiency of the *NSD1* gene (5q35) which encodes nuclear receptor-binding SET Domain protein 1, a lysine-specific histone methyltransferase that has a critical role in epigenetic regulation [3,4,5]. Sotos syndrome-associated *NSD1* variants are most commonly truncating intragenic variants although microdeletions occur in ~10% of cases and missense variants in about a quarter [5]. The clinical phenotype of Sotos syndrome is variable, and the differential diagnosis can include other overgrowth–intellectual disability (OGID) conditions such as Weaver syndrome (MIM#277590), Tatton-Brown–Rahman syndrome (MIM#615879), Malan syndrome (MIM#614753), *NF1A* haploinsufficiency, Simpson–Golabi–Behmel syndrome (MIM#312870), and Luscan–Lumish syndrome (MIM#616831) [6,7,8,9,10,11].

Identification of a pathogenic variant in *NSD1* enables differentiation of a case of Sotos syndrome from other potential OGID diagnoses [5,12]. However, the detection of a rare *NSD1* variant may not clarify the diagnosis if the variant is classified as a variant of uncertain significance (VUS). Recently, it has been recognised that specific blood DNA methylation patterns (‘episignatures’) may be associated with developmental disorders that are caused by pathogenic variants in genes that encode epigenetic regulators, such as *NSD1* [13,14,15,16,17,18]. Pathogenic variants in *NSD1* are associated with an episignature that is characterised by CpG hypomethylation and this finding may be used to resolve VUSs in *NSD1* to pathogenic or benign categories [13,19].

Familial cases of Sotos syndrome are rare but intrafamilial variability in phenotype has been noted in family members with identical *NSD1* pathogenic variants, and it has been suggested that stochastic and epigenetic, and environmental factors and genetic modifiers might influence the phenotype of Sotos syndrome [5]. Recently, we have identified a family with mild Sotos syndrome and intrafamilial phenotypic variability associated with a germline *NSD1* pathogenic variant. We then investigated the relationship between clinical and epigenetic variability between the three affected relatives.

## 2. Materials and Methods

### 2.1. Patient Samples and Clinical Measurements

We studied 11 individuals with germline variants in *NSD1*. Eight unrelated participants (mean age: 9.3 years, range: 2 to 31 years), had been diagnosed with germline pathogenic intragenic variants in the *NSD1* gene and three cases (aged: 0.5, 18, and 54 years) from a single family had been diagnosed with an *NSD1* exon 3 deletion. Informed written consent was obtained for all participants, and ethical approval was provided by the South Birmingham Research Ethics Committee (Molecular Pathology of Human Genetic Disease study). The reported investigations were conducted in accordance with the principles outlined in the Declaration of Helsinki (1975, revised in 2013). We used centile charts for assessment of height and head circumference from the Child Growth Foundation (published in Firth and Hirst [20]).

### 2.2. Molecular Genetic Analysis

Genomic DNA was extracted using standard methods in various regional genetics laboratories (RGLs). Microarray analysis was performed in an NHS diagnostic laboratory with the Oxford Gene Technology (OGT) CytoSure 8 × 60 k Constitutional v3 design array platform. Three consecutive probes are required to call a deletion. Identification and classification of germline *NSD1* variants was performed by RGLs according to local protocols. DNA methylation profiling was performed as described previously by EPIC-NGS pipeline [21,22]. In brief, DNA samples were sequenced on an Illumina NextSeq 2000 platform using the TruSeq^®^ Methyl Capture EPIC kit (Illumina, San Diego, CA, USA). Bisulfite conversion, library preparation, target enrichment, and sequencing were carried out at the Stratified Medicine Core Laboratory, Department of Medical Genetics, University of Cambridge, in accordance with the previously described procedure [21].

### 2.3. Bioinformatic Analysis

Sequencing data were obtained in the FastQ format, and pre-processing steps, including adapter trimming, quality control, and alignment to the GRCh37 reference genome, were performed using an in-house pipeline, with PCR duplicates removed. Methylation data was extracted using the Bismark methylation extractor, and CpG methylation values, *p*-values, and CpG region annotations were generated using the RnBeads package (https://rnbeads.org/) in R (version 3.6.3). Pre-processing also involved the exclusion of sex chromosomes and CpG sites near SNP regions, applying a minimum coverage threshold of 10×.

Differential methylation analysis was performed using the ‘limma’ method in RnBeads. Batch effects (age, gender, and batch) were assessed via principal component analysis (PCA) and adjusted with surrogate variable analysis (SVA) if significant. To account for cell type heterogeneity, adjustments were made using the Houseman method [23]. Methylation site *p*-values were calculated using a two-sided Welch’s *t*-test or linear models, and combined *p*-values for CpG islands were obtained through Fisher’s method.

For differential methylation block (DMB) analysis, CpG sites or CpG islands with neighbouring CpGs spaced more than 5 kb apart were selected. Neighbouring CpGs in shore, shelf, and open sea regions were merged and classified as DMBs, which were further grouped based on functional similarities. DMBs ranged in size from 5 to 200 kb. CpG islands were defined using the Ensembl genome browser (http://www.ensembl.org) [24]. Significant DMBs were identified by excluding those with fewer than 3 EPIC-NGS target regions and selecting only those with an adjusted *p*-value (FDR) below 0.05 and a methylation difference greater than 20% between control and disease groups. Data visualisation was performed using the ComplexHeatmap R package (version 2.20), applying hierarchical clustering with the complete-linkage method. All beta-values were Z-score normalised and compared to healthy controls (*n* = 64, in-house healthy patient samples, confirmed as free from any specific methylation episignatures through randomised control analysis).

## 3. Results

### 3.1. Clinical and Genetic Findings in a Family with a NSD1 Exon 3 Deletion

Three members, the proband, her mother, and maternal grandfather with a *NSD1* exon 3 deletion were identified:

Patient 1 (III:1): The proband was the first child of unrelated white British parents. She was born prematurely at 34 weeks and 5 days gestation and required high flow oxygen for transient tachypnoea of the newborn. She developed neonatal jaundice treated with phototherapy and initially required nasogastric feeds. A cranial ultrasound scan identified mild dilatation of the third and lateral ventricles and clubbing of the anterior horns.

At eight months of age, she developed infantile spasms with hypsarrhythmia on EEG. Her seizures were resolved with treatment with ACTH and vigabatrin. Subsequent medical problems included recurrent urinary tract infections, constipation, and recurrent upper respiratory tract and ear infections. Renal ultrasound scan identified nephrocalcinosis. She achieved her gross motor milestones at typical ages and walked at the age of 14 months; however, speech delay was evident, with single words at the age of 2 years and short phrases by the age of 4 years. She displayed hyperactive and challenging behaviour and was under assessment for possible autism spectrum disorder and attention deficit hyperactivity disorder (ADHD).

**Figure 1:**
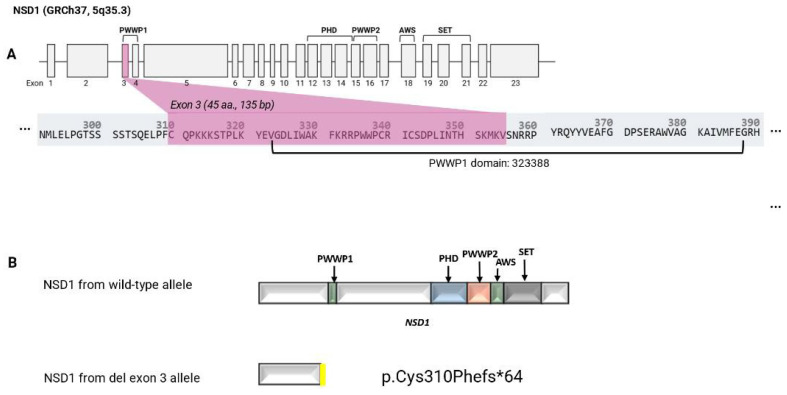
Exon structure of *NSD1* and predicted effect of the deletion (**A**): Exon structure of *NSD1*, highlighting exon 3, which was included in the genomic deletion identified by microarray and confirmed by MLPA (**B**): Predicted effect of the deletion at Chr5:176618784_176619062 (NM_022455.4, GRCh37) that removes exon 3 of *NSD1* and results in a frameshift. The top panel shows the predicted NSD1 translation product from the wild-type allele and the lower panel the predicted truncated NSD1 protein (p.Cys310Phefs*64) from the exon 3 deletion allele (in the absence of nonsense-mediated mRNA decay).

Growth parameters were within the normal range. At nine weeks of age, her weight was 3.93 kg (26th centile) and head circumference 36.7 cm (48th centile). At the age of two her head circumference measured 47.5 cm (7th centile) and at age three her height was 100.5 cm (61st centile) and weight 14.8 kg (45th centile). Clinical assessment at age 12 months and 4 years showed a lack of the characteristic facial features of Sotos syndrome. Microarray analysis was performed in infancy and revealed a microdeletion at 5q35.3 (176618784- 176619062del (GRCh37)) (arr[hg19] 5q35.5 (17661874_176619062) × 1) containing exon 3 of *NSD1* (Figure 1A). The coding sequence of *NSD1* is represented in 23 exons and the reference *NSD1* transcript (NM_022455.4, GRCh37) encodes a full-length protein of 2696 amino acids. The deletion of exon 3 was reported according to HGVS nomenclature as NM_022455.4 *NSD1* c.(921+1_928-1)_(1063+1_1064-1), (see https://hgvs-nomenclature.org/stable/recommendations/DNA/deletion/, accessed 23 September 2025) as the exact breakpoints were not defined but exon 2 and 4 were not shown to be deleted by MLPA and microarray analysis. The exon 3 deletion was predicted to lead to an out-of-frame deletion resulting in a truncated *NSD1* gene product (p.(Cys310Phefs*64)). The truncated NSD1 protein, in the absence of nonsense-mediated decay, lacked multiple NSD1 function domains (PWWP1, PHD, PWWP2, AWS, SET) (see Figure 1B).

Patient 2 (II:1): Following the detection of the *NSD1* exon 3 deletion in the proband, parental studies using MLPA confirmed that the 5q35 microdeletion was maternally inherited. The proband’s mother was aged 18 years at the time of her daughter’s birth. Her own clinical history was of preterm delivery at 33 weeks by emergency Caesarean section for maternal pre-eclampsia. She was reported to weigh 1.8 kg at birth (35th centile) and was on the neonatal unit for four weeks for feeding difficulties and treatment of jaundice.

As a young child she was diagnosed with spastic cerebral palsy. She had frequent ear infections and had an insertion of grommets and a tonsillectomy. She first walked and spoke her first words at 18 months of age. She attended mainstream school with 1:1 help and passed a limited number of school examinations at the age of 16. As a child she was described as being accident-prone and having unusual behaviours, including no sense of danger and a very high pain threshold. Her growth parameters were in the normal range with a height of 162.7 cm (44th centile) and head circumference 57.5 cm (91st–98th) centile [20]. On review in the genetics clinic aged 19 years, she had a slightly long face but her features were not considered characteristic of Sotos syndrome.

Patient 3 (I:1): Following the identification of the 5q35 deletion in Patient 2 (mother of the proband), parental studies (*NSD1* MLPA) identified the deletion to be paternally inherited. Her father (grandfather of the proband) was reviewed in clinic aged 54 years. He was born at term with a reported birthweight of 4.5 kg (98th centile) and had neonatal jaundice. He reported being ‘clumsy’ as a child and received additional help at school. He had mild scoliosis. As an adult he had chronic bronchitis, asthma, and cervical and lumbar disc prolapse with radiculopathy. He was 188 cm tall (94th centile) with a head circumference of 60.1 cm (91st–98th centile) [20].

### 3.2. DNAm Episignature Analysis

#### Comparison of Genome-Wide Methylation Profiles in Sotos Syndrome and Individuals with a *NSD1* Exon 3 Deletion

Methylation profiling was undertaken on DNA from eight individuals with Sotos syndrome and a confirmed intragenic pathogenic variant in *NSD1* (seven patients with truncating variants and one missense variant; see Table 1) and three familial carriers of the *NSD1* exon 3 deletion using a next generation sequencing based assay of genome wide CpG methylation for >3M CpGs [25]. Comparison of the DNA methylation (DNAm) profiles for the 8 individuals with Sotos syndrome to 64 healthy control samples identified (after adjustments for age, sex, and blood composition) 8487 differentially methylated positions (DMPs), including 718 CpG islands. These DMPs were selected on the basis of a methylation difference of 20% and a significant adjusted *p*-value of 0.05 (FDR < 0.05). 99.6% (8461/8487) of the DMPs identified demonstrated hypomethylation in the individuals with Sotos syndrome (see Supplementary Figure S1 and Supplementary Table S1).

**Figure 2:**
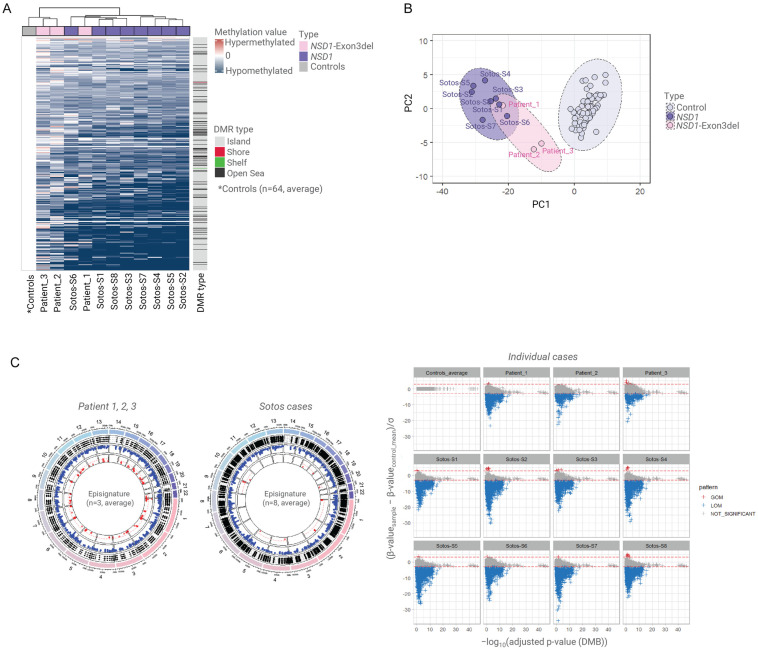
Methylation episignatures for Sotos syndrome and *NSD1* exon 3 deletion familial cases. (**A**). The methylation episignature for confirmed Sotos cases (*n* = 8) and familial patients with *NSD1* exon deletion (*n* = 3) compared to controls (*n* = 64). A total of 6728 differentially methylated positions (DMPs), including CpG islands and DMBs, were identified. Patient 1 (proband) was classified within the pathogenic *NSD1* group, while the parental samples (Patients 2 and 3) were classified closer to the control group, exhibiting a milder episignature. (**B**). Principal component analysis (PCA) clustering demonstrated that all *NSD1* cases, including exon 3 deletion cases, were distinct from the control group. Patient 1 was more closely related to the Sotos group than Patients 2 and 3. (**C**). Among the 6728 significant DMPs, hypomethylation was predominantly observed in the patient group. However, Patients 2 and 3 exhibited a milder degree of hypomethylation compared to Patient 1 and the other *NSD1* cases.

We then compared the DNAm profiles for the three familial *NSD1* exon 3 deletion cases to the confirmed cases of Sotos syndrome (*n* = 8) and control samples (*n* = 64). The familial cases (Patients 1, 2, and 3) all showed hypomethylation but there were variations between the degree of hypomethylation among the three individuals (Figure 2). In an unsupervised cluster analysis of all 75 samples, the proband (Patient 1) was grouped with the 8 confirmed cases of Sotos syndrome, while her mother (Patient 2) and grandfather (Patient 3) were grouped between the controls and the samples from confirmed cases of Sotos syndrome (Figure 2).

In addition, a total of 1537 statistically significant DMPs (FDR < 0.05) were detected between the deletion carriers (Patients 1, 2, and 3) and normal controls (*n* = 64), and >85% of these DMPs (1320/1537) overlapped with the DMPs identified between the Sotos syndrome cohort (confirmed cases, *n* = 8) and controls (*n* = 64) (DMP = 8487) (Supplementary Figure S1 and Supplementary Table S2). This finding suggested that allelic heterogeneity in *NSD1* can be associated with variability in the extent of genome DNAm alterations and these might correlate with the clinical phenotype.

### 3.3. Gene Pathway Analysis of Differentially Methylated Regions

To assess the potential functional relevance of differentially methylated regions, we identified DMBs (and their associated genes) that were significantly altered in *NSD1* exon 3 deletion cases (*n* = 3) and/or participants with Sotos syndrome (*n* = 8). We extracted DMPs (including those within CpG islands and open sea regions) and identified genes that were associated with altered DMBs in only in the exon 3 deletion group (*n* = 10 genes), only in the Sotos syndrome group (*n* = 400) and those altered in both groups (*n* = 60) (see Supplementary Table S3).

Gene ontology (GO) term analysis (https://geneontology.org/) was performed after excluding unmapped IDs (gene/protein identifiers that could not be linked to annotation databases) and compared between the three groups (see Supplementary Figure S3). We found that binding (GO:0005488) and catalytic activity (GO:0003824) were the most frequently enriched terms within the molecular function (MF) category, while biological regulation (GO:0065007), cellular process (GO:0009987), and developmental process (GO:0032502) were the most prominent terms within the biological process (BP) category. The distribution of GO term representation showed broadly consistent patterns across all three groups (see Supplementary Figure S3).

We also performed functional profiling using g:Profiler (https://biit.cs.ut.ee/gprofiler/gost, accessed 27 August 2025). Databases selected included KEGG, Reactome (REAC), and WikiPathways (WP) for biological pathways. Furthermore, Human Protein Atlas (HPA), CORUM, and Human Phenotype Ontology (HPO) were used for protein and phenotype associations. This approach identified thirteen statistically significant terms (Benjamini–Hochberg FDR < 0.05), including HP (*n* = 5), KEGG (*n* = 1), and REAC (*n* = 7) in the exon 3 deletion carriers’ cohort (*n* = 3) (see Supplementary Figure S4 and Supplementary Table S4). In comparison, the Sotos-only cohort (*n* = 8) showed significant enrichment in CORUM (*n* = 95) and TF (*n* = 133), whereas the overlapping group (exon 3 deletion and Sotos syndrome) revealed associations with CORUM (*n* = 19) and HPA (*n* = 5).

In addition, to investigate disease associations, we performed an analysis using Gene2Phenotype (https://www.ebi.ac.uk/gene2phenotype/, accessed 5 September 2025), in which we identified developmental disorder genes that were associated with altered DMBs and noted overlapping HPO terms between Sotos syndrome and the syndromes associated with the selected developmental disorder genes (see Supplementary Table S5). HPO terms commonly associated with Sotos syndrome including tall stature (HP:0000098), macrocephaly (HP:0000256), developmental delay (HP:0001263), frontal bossing (HP:0002007), downslanted palpebral fissures (HP:0000494), joint laxity (HP:0001388), scoliosis (HP:0002650), and neonatal hypotonia (HP:0001319) were observed in genes in the Sotos-only group.

## 4. Discussion

We investigated a three-generation family in which three members (proband, mother, and grandfather) harboured an exon 3 deletion in *NSD1* that was predicted to cause a truncated NSD1 protein and would, therefore, like other loss-of-function *NSD1* variants, be associated with a Sotos syndrome phenotype [4,5]. However, Sotos syndrome was not included in the initial clinical differential diagnosis though the clinical features and imaging findings in Patient 1, including ventricular dilatation on cranial ultrasound scan, infantile spasms, and nephrocalcinosis, have been previously reported in Sotos syndrome [5,26]. The history of maternal pre-eclampsia, neonatal feeding difficulties and jaundice, and childhood tendency to upper respiratory tract and ear infections in both Patient 1 and 2 are non-specific but common features of Sotos syndrome [27].

Furthermore, the developmental and behavioural phenotypes reported in all three family members are also consistent with a diagnosis of Sotos syndrome. However, the characteristic facial features and overgrowth associated with Sotos syndrome were not present in any of the three individuals. Although the clinical features of Sotos syndrome can be highly variable between individuals, and tall stature is not universal, characteristic facial features and macrocephaly are cardinal features of this disorder [5] and so the finding of an intragenic *NSD1* deletion in this family was considered unexpected and their phenotypes classed as atypical.

Sotos syndrome is associated with a wide range of intragenic variants and deletions of the *NSD1* gene, though genotype–phenotype associations are not prominent. A tendency for the patients with whole gene deletions to have more significant intellectual disability and less pronounced overgrowth than patients with intragenic variants has been reported but the full spectrum of features is seen in both subgroups and there are no confirmed genotype–phenotype associations for *NSD1* intragenic variants [5]. In a previous case report of a patient with a mosaic deletion of part of intron 2 and the entire exon 3 of *NSD1*, the patient had an atypical phenotype with an absence of characteristic facial features of Sotos syndrome and normal growth parameters, though he did have a developmental delay and learning disability (including severe speech delay), muscular hypotonia, and cryptorchidism consistent with Sotos syndrome [28]. In that case the atypical clinical features were attributed to mosaicism for the deletion [28]. Review of the MLPA results in the grandfather (and other relatives) demonstrated no evidence of mosaicism in the family reported here. However, our findings suggest the possibility that *NSD1* exon 3 deletions might be associated with an atypical phenotype that includes the neurodevelopmental and some medical features of Sotos syndrome, but not the characteristic facial features or overgrowth. Though it is possible that the deletion might have affected splicing of differentially expressed NSD1 transcripts, we were unable to test this hypothesis.

DNA methylation episignatures in developmental disorders are heterogeneous with respect to the degree of methylation alterations, the types of alterations (hypo- and hypermethylation), and the positions of the DMPs [16,21,22,29]. Individuals with classical Sotos syndrome have a distinctive episignature with a large number of DMPs that overwhelmingly demonstrate hypomethylation compared to control samples [13]. Although the proband and her relatives did not show some classical features of Sotos syndrome (macrocephaly and facial features), the genomic methylation alterations in the proband resembled those in Sotos syndrome with many DMPs that demonstrated loss of methylation (Figure 2C). Nevertheless, the DMPs in the proband overlapped very substantially with those in the confirmed Sotos syndrome patients. The mother and grandfather of the proband carried the same *NSD1* exon 3 deletion as the proband but clinically appeared to be less severely affected and, interestingly, the methylation episignature in the mother and grandfather were, whilst distinct from the unaffected controls, also milder than in the more severely affected proband. This raises the intriguing possibility that methylation episignatures in Sotos syndrome might correlate with clinical phenotype and, if so, might have potential utility not only in variant interpretation and diagnostics but also in prognostication. Previously, we identified that clear epigenetic differences between patients with *SETD2* variants causing Luscan–Lumish and Rabin–Pappas syndromes [22] and others have demonstrated that in Menke–Hennekam syndrome three subtypes were delineated according to the location of pathogenic variants in three domains of EP300 and CREBBP and the subtypes showed phenotypic and mild episignature differences [30]. In addition, missense variants in exons 38 or 39 of *KMTD2* are associated with a multiple malformations disorder that is clinically and epigenetically distinct from Kabuki syndrome type 1 [31]. However, there are relatively few examples of genotype–epigenotype–phenotype correlations in chromatinopathies as numbers of patients, allelic heterogeneity, and phenotypic variability might hinder such studies. Whilst familial cases of developmental disorders caused by haploinsufficiency of epigenetic regulators are infrequent, when they do occur, they can offer an opportunity to look for epigenotype–phenotype correlations in family members with an identical germline variant. Furthermore, variations in the extent of methylation changes may be easier to study in developmental disorders with prominent episignatures (e.g., Sotos syndrome) than in those with smaller differences from controls. Additionally, NGS-based assays that can analyse many more CPGs than methylation array-based assays may increase the likelihood of detecting significant correlations.

## Figures and Tables

**Table 1:** Variant details for 8 Sotos syndrome patients and three familial carriers of the *NSD1* exon 3 deletion.

Sample ID	Type	Variant Type	Sex; Age
Sotos-S1	Sotos Syndrome	c.5791T > C p.(Cys931Arg) de novo	M: 8
Sotos-S2	Sotos Syndrome	pathogenic frameshift variant c.5278_5282delTCTG p.(Val1760Glyfs*2) exon 15	F: 2
Sotos-S3	Sotos Syndrome	pathogenic frameshift variant c.1187delC p.(Pro396Leufs*23) exon 4	M: 9
Sotos-S4	Sotos Syndrome	likely pathogenic de novo duplica- tion exons 11-22	F: 15
Sotos-S5	Sotos Syndrome	c.1633dupA,p.Thr545AsnfsTer5 pathogenic de novo	M: 0.7
Sotos-S6	Sotos Syndrome	c.6454C > T p.(Arg2152Ter)	M: 6
Sotos-S7	Sotos Syndrome	c.4072C > T p.(Gln1358Ter)	M: 31
Sotos-S8	Sotos Syndrome	c.5982dupT p.(Asn1995Ter)	M: 2
Patient 2 (II:1)	Mild-Sotos NSD1 Developmental Disorder	NSD1 Exon 3 deletion	F: 18
Patient 1 (III:1)	Mild-Sotos NSD1 Developmental Disorder Sotos	NSD1 Exon 3 deletion	F: 0.1
Patient 3 (I:1)	Mild-Sotos NSD1 Developmental Disorder Sotos	NSD1 Exon 3 deletion	M: 54

## Data Availability

The patient methylation profiling datasets analysed during the current study are not freely available because of ethical restrictions but requests should be made to the corresponding author.

## Supplementary Materials

The following supporting information can be downloaded at https://www.mdpi.com/article/10.3390/genes16101190/s1, Supplementary Figure S1: Methylation episignature for individuals with Sotos syndrome with confirmed *NSD1* variants (*n* = 8). A total of 8487 DMPs were identified as hypomethylated. Of these, 3373 (approximately 40%) were significantly hypomethylated, exceeding three standard deviations (3SD) from the healthy control group. No DMPs demonstrated a gain of methylation exceeding 3SD. Supplementary Figure S2: Methylation episignature for the familial patient group with *NSD1* exon 3 deletions (*n* = 3). A total of 1537 significant DMPs were identified in the control group. Patient 1 showed 551 out of 1537 DMPs as hypomethylated, while Patients 2 and 3 exhibited an average of 251 out of 1537 hypomethylated DMPs. No DMPs exhibited a gain of methylation exceeding the 3SD compared to the control group. Supplementary Figure S3: Gene Ontology (GO) term analysis, after excluding unmapped IDs, for the three groups of genes (those altered in exon 3 deletion cohort, Sotos-only cohort, and both groups). Supplementary Figure S4: Detailed functional profiling by g:Profiler including significant terms and adjusted *p*-values. Supplementary Table S1: Significant differentially methylated positions (DMPs) (Sotos syndrome patients *n* = 8). Supplementary Table S2: Overlapping of differentially methylated positions (DMPs) between Sotos syndrome patients (*n* = 8) and deletion carriers (*n* = 3). Supplementary Table S3: Pathway analysis of DMB-associated genes with G2P-linked disease annotations. Supplementary Table S4: Detailed functional profiling by g:Profiler including significant terms and adjusted *p*-values. Supplementary Table S5: Genes for inherited developmental disorders associated with regions (DMBs) showing significantly altered methylation in individuals with a familial exon 3 deletion (3 individuals; Exon3delonly), classical Sotos syndrome (*n* = 8 individuals; Sotos only), and in both groups (*n* = 11; both groups). Information on developmental disorder genes was accessed at the Gene2Phenotype resource (https://www.ebi.ac.uk/gene2phenotype/) (accessed on 3 September 2025). HPO (Human Phenotype Ontology) terms (when present in Gene2Phenotype) are recorded for each condition, those HPO terms highlighted in red are recorded for both Sotos syndrome and the relevant condition. Relevant HPO terms for frequent features of Sotos syndrome include tall stature (HP:0000098), macrocephaly (HP:0000256), developmental delay (HP:0001263), frontal bossing (HP:0002007), downslanted palpebral fissures (HP:0000494), joint laxity (HP:0001388), scoliosis (HP:0002650), and neonatal hypotonia (HP:0001319).

## Abbreviations

The following abbreviations are used in this manuscript:

SS	Sotos syndrome
OGID	Overgrowth–intellectual disability
VUS	Variant of uncertain significance
PCA	Principal component analysis
SVA	Surrogate variable analysis
DMB	Differential methylated block
ADHD	Attention deficit hyperactivity disorder

## References

[1] Sotos J.F., Dodge P.R., Muirhead D., Crawford J.D., Talbot N.B. (1964). Cerebral gigantism in childhood A syndrome of excessively rapid growth with acromegalic features and a nonprogressive neurologic disorder. N. Engl. J. Med..

[2] Ocansey S., Cole T.R.P., Rahman N., Tatton-Brown K., Adam M.P., Feldman J., Mirzaa G.M., Pagon R.A., Wallace S.E., Amemiya A. (1993). Sotos Syndrome. GeneReviews^®^.

[3] Kurotaki N., Imaizumi K., Harada N., Masuno M., Kondoh T., Nagai T., Ohashi H., Naritomi K., Tsukahara M., Makita Y. (2002). Haploinsufficiency of NSD1 causes Sotos syndrome. Nat. Genet..

[4] Douglas J., Hanks S., Temple I.K., Davies S., Murray A., Upadhyaya M., Tomkins S., Hughes H.E., Cole R.T., Rahman N. (2003). NSD1 Mutations Are the Major Cause of Sotos Syndrome and Occur in Some Cases of Weaver Syndrome but Are Rare in Other Overgrowth Phenotypes. Am. J. Hum. Genet..

[5] Tatton-Brown K., Douglas J., Coleman K., Baujat G., Cole T.R., Das S., Horn D., Hughes H.E., Temple I.K., Faravelli F. (2005). Genotype-phenotype associations in Sotos syndrome: An analysis of 266 individuals with NSD1 aberrations. Am. J. Hum. Genet..

[6] Cole T.R., Dennis N.R., Hughes H.E. (1992). Weaver syndrome. J. Med. Genet..

[7] Li M., Shuman C., Fei Y.L., Cutiongco E., Bender H., Stevens C., Wilkins-Haug L., Day-Salvatore D., Yong S., Geraghty M. (2001). GPC3 Mutation Analysis in a Spectrum of Patients with Overgrowth Expands the Phenotype of Simpson-Golabi-Behmel Syndrome. Am. J. Med. Genet..

[8] Luscan A., Laurendeau I., Malan V., Francannet C., Odent S., Giuliano F., Lacombe D., Touraine R., Vidaud M., Pasmant E. (2014). Mutations in SETD2 cause a novel overgrowth condition. J. Med. Genet..

[9] Malan V., Rajan D., Thomas S., Shaw A.C., Picard H.L.D., Layet V., Till M., van Haeringen A., Mortier G., Nampoothiri S. (2010). Distinct effects of allelic NFIX mutations on nonsense-mediated mRNA decay engender either a sotos-like or a Marshall-Smith Syndrome. Am. J. Hum. Genet..

[10] Tatton-Brown K., Seal S., Ruark E., Harmer J., Ramsay E., Del Vecchio Duarte S., Zachariou A., Hanks S., O’Brien E., Aksglaede L. (2014). Mutations in the DNA methyltransferase gene DNMT3A cause an overgrowth syndrome with intellectual disability. Nat. Genet..

[11] Lu W., Quintero-Rivera F., Fan Y., Alkuraya F.S., Donovan D.J., Xi Q., Turbe-Doan A., Li Q.-G., Campbell C.G., Shanske A.L. (2007). NFIA Haploinsufficiency Is Associated with a CNS Malformation Syndrome and Urinary Tract Defects. PLoS Genet..

[12] Baujat G., Rio M., Rossignol S., Sanlaville D., Lyonnet S., Le Merrer M., Munnich A., Gicquel C., Cormier-Daire V., Colleaux L. (2004). Paradoxical NSD1 Mutations in Beckwith-Wiedemann Syndrome and 11p15 Anomalies in Sotos Syndrome. Am. J. Hum. Genet..

[13] Choufani S., Cytrynbaum C., Chung B.H.Y., Turinsky A.L., Grafodatskaya D., Chen Y.A., Cohen A.S.A., Dupuis L., Butcher D.T., Siu M.T. (2015). NSD1 mutations generate a genome-wide DNA methylation signature. Nat. Commun..

[14] Aref-Eshghi E., Bend E.G., Hood R.L., Schenkel L.C., Carere D.A., Chakrabarti R., Nagamani S.C.S., Cheung S.W., Campeau P.M., Prasad C. (2018). BAFopathies’ DNA methylation epi-signatures demonstrate diagnostic utility and functional continuum of Coffin–Siris and Nicolaides–Baraitser syndromes. Nat. Commun..

[15] Aref-Eshghi E., Kerkhof J., Pedro V.P., Barat-Houari M., Ruiz-Pallares N., Andrau J.-C., Lacombe D., Van-Gils J., Fergelot P., Dubourg C. (2020). Evaluation of DNA Methylation Episignatures for Diagnosis and Phenotype Correlations in 42 Mendelian Neurodevelopmental Disorders. Am. J. Hum. Genet..

[16] Levy M.A., McConkey H., Kerkhof J., Barat-Houari M., Bargiacchi S., Biamino E., Bralo M.P., Cappuccio G., Ciolfi A., Clarke A. (2022). Novel diagnostic DNA methylation episignatures expand and refine the epigenetic landscapes of Mendelian disorders. HGG Adv..

[17] Levy M.A., Relator R., McConkey H., Pranckeviciene E., Kerkhof J., Barat-Houari M., Bargiacchi S., Biamino E., Bralo M.P., Cappuccio G. (2022). Functional correlation of genome-wide DNA methylation profiles in genetic neurodevelopmental disorders. Hum. Mutat..

[18] Sadikovic B., Levy M.A., Kerkhof J., Aref-Eshghi E., Schenkel L., Stuart A., McConkey H., Henneman P., Venema A., Schwartz C.E. (2021). Clinical epigenomics: Genome- wide DNA methylation analysis for the diagnosis of Mendelian disorders. Genet. Med..

[19] Ferilli M., Ciolfi A., Pedace L., Niceta M., Radio F.C., Pizzi S., Miele E., Cappelletti C., Mancini C., Galluccio T. (2022). Genome-Wide DNA Methylation Profiling Solves Uncertainty in Classifying NSD1 Variants. Genes.

[20] Firth H.V., Hurst J.A. (2017). Oxford Desk Reference: Clinical Genetics and Genomics.

[21] Lee S., Ochoa E., Barwick K., Cif L., Rodger F., Docquier F., Pérez-Dueñas B., Clark G., Martin E., Banka S. (2022). Comparison of methylation episignatures in KMT2B-and KMT2D-related human disorders. Epigenomics.

[22] Lee S., Menzies L., Hay E., Ochoa E., Docquier F., Rodger F., Deshpande C., Foulds N.C., Jacquemont S., Jizi K. (2023). Epigenotype–genotype–phenotype correlations in SETD1A and SETD2 chromatin disorders. Hum. Mol. Genet..

[23] Houseman E.A., Accomando W.P., Koestler D.C., Christensen B.C., Marsit C.J., Nelson H.H., Wiencke J.K., Kelsey K.T. (2012). DNA methylation arrays as surrogate measures of cell mixture distribution. BMC Bioinform..

[24] Müller F., Scherer M., Assenov Y., Lutsik P., Walter J., Lengauer T., Bock C. (2019). RnBeads 20: Comprehensive analysis of DNA methylation data. Genome Biol..

[25] Illumina (2016). TruSeq Methyl Capture EPIC Library Prep Kit. Illumina.

[26] Kenny J., Lees M.M., Drury S., Barnicoat A., Hoff W.V., Palmer R., Morrogh D., Waters J.J., Lench N.J., Bockenhauer D. (2011). Sotos syndrome, infantile hyper-calcemia, and nephrocalcinosis: A contiguous gene syndrome. Pediatr. Nephrol..

[27] Baujat G., Cormier-Daire V. (2007). Sotos syndrome. Orphanet J. Rare Dis..

[28] Castronovo C., Rusconi D., Crippa M., Giardino D., Gervasini C., Milani D., Cereda A., Larizza L., Selicorni A., Finelli P. (2013). A novel mosaic NSD1 intra- genic deletion in a patient with an atypical phenotype. Am. J. Med. Genet. A.

[29] Lee S., Ochoa E., Badura-Stronka M., Donnelly D., Lederer D., Lynch S.A., Gardham A., Morton J., Stewart H., Docquier F. (2023). Germline pathogenic variants in HNRNPU are associated with alterations in blood methylome. Eur. J. Hum. Genet..

[30] Haghshenas S., Bout H.J., Schijns J.M., Levy M.A., Kerkhof J., Bhai P., McConkey H., Jenkins Z.A., Williams E.M., Halliday B.J. (2024). Menke-Hennekam syndrome; delineation of domain-specific subtypes with distinct clinical and DNA methylation profiles. HGG Adv..

[31] Cuvertino S., Hartill V., Colyer A., Garner T., Nair N., Al-Gazali L., Canham N., Faundes V., Flinter F., Hertecant J. (2020). A restricted spectrum of missense KMT2D variants cause a multiple malformations disorder distinct from Kabuki syndrome. Genet. Med..

