# Supplementary material for: Familial NSD1 Exon 3 Deletion Associated with Phenotypic and Epigenetic Variability
Source: Genes (Basel). 2025 Oct 13;16(10):1190. doi: 10.3390/genes16101190 (PMC12563888; doi:10.3390/genes16101190)
Supplement: Supplementary file 1 [file genes-16-01190-s001.zip › SUPPLEMENTARY DATA for Lee et al-combined-combined 05092025.pdf]

SUPPLEMENTARY DATA

Familial *NSD1* Exon 3 Deletion Associated with Phenotypic and Epigenetic Variability

Sunwoo Liv Lee [1], Alison Foster [2,3], Dalit May [1], Ciara Batterton [4], Eguzkine Ochoa [1], Bryndis Yngvadottir [1], Ruth Armstrong [5], Meena Balasubramanian [6], Mary O'Driscoll [2], Marc Tischkowitz [5], France Docquier [1,7], Fay Rodger [1,7], Ezequiel Martin [1,7], Ana Toribio [1,7] and Eamonn R Maher [1,2,5,8,*]

1 Department of Genomic Medicine, University of Cambridge, Cambridge CB2 0QQ, UK
2 Clinical Genetics Unit, Birmingham Women's and Children's NHS Trust, Birmingham B15 2TG, UK
3 Peninsula Clinical Genetics, Royal Devon and Exeter NHS Trust, Exeter, UK.
4 West Midlands Genomics Laboratory, Birmingham Women's and Children's NHS Trust, Birmingham, B15 2TG, UK
5 Department of Clinical Genetics, Cambridge University Hospitals NHS Trust, Cambridge CB2 0QQ, UK
6 Division of Clinical Medicine, University of Sheffield, Sheffield, UK and Sheffield Clinical Genetics Service, Sheffield Children's NHS Foundation Trust, Sheffield, UK.
7 Stratified Medicine Core Laboratory NGS Hub, Cambridge Biomedical Campus, Cambridge, CB20QQ, UK
8 Aston Medical School, College of Health and Life Sciences, Birmingham B7 4ET, UK
* Correspondence: e.maher@aston.ac.uk

Contents:

Supplementary Figures

Supplementary Figure 1: Methylation episignature for individuals with Sotos syndrome with confirmed *NSD1* variants (n=8). A total of 8,487 DMPs were identified as hypomethylated. Of these 3,373 (approximately 40%) were significantly hypomethylated, exceeding three standard deviations (3SD) from the healthy control group. No DMPs demonstrated a gain of methylation exceeding 3SD.

Supplementary Figure 2: Methylation episignature for the familial patient group with *NSD1* exon 3 deletions (n=3). A total of 1,537 significant DMPs were identified control group. Patient 1 showed 551 out of 1,537 DMPs as hypomethylated, while Patients 2 and 3 exhibited an average of 251 out of 1,537 hypomethylated DMPs. No DMPs exhibited a gain of methylation exceeding the 3SD compared to the control group.

Supplementary Figure 3: Gene Ontology (GO) term analysis, after excluding unmapped IDs, for the three groups of genes (those altered in Exon 3 deletion cohort, Sotos only cohort, and both groups)

Supplementary Figure 4: Detailed functional profiling by g:Profiler including significant terms and adjusted p-values

Supplementary Tables

Supplementary Table S1: Significant differentially methylated positions (DMPs) (Sotos syndrome patients n=8)

Supplementary Table S2: Overlapping differentially methylated positions (DMPs) between Sotos syndrome patients (n=8) and deletion carriers (n=3)

Supplementary Table S3: Pathway analysis of DMB-associated genes with G2P-linked disease annotations

Supplementary Table S4: Detailed functional profiling by g:Profiler including significant terms and adjusted p-values

Supplementary Table S5: Genes for inherited developmental disorders associated with regions (DMBs) showing significantly altered methylation in individuals with a familial exon 3 deletion (3 individuals; Exon3delonly), classical Sotos syndrome (n=8 individuals; Sotos only) and in both groups (n=11; Both groups). Information on developmental disorder genes was accessed at the Gene2Phenotype resource (https://www.ebi.ac.uk/gene2phenotype/) (accessed 3rd September 2025). HPO (Human Phenotype Ontology) terms (when present in Gene2Phenotype) are recorded for each condition those HPO terms highlighted in red are recorded for both Sotos syndrome and the relevant condition. Relevant HPO terms for frequent features of Sotos syndrome include: tall stature (HP:0000098), macrocephaly (HP:0000256), developmental delay (HP:0001263), Frontal bossing (HP:0002007), Downslanted palpebral fissures (HP:0000494), joint laxity (HP:0001388), scoliosis (HP:0002650), neonatal hypotonia (HP:0001319)

Supplementary Figure 1: Methylation episignature for individuals with Sotos syndrome with confirmed *NSD1* variants (n=8). A total of 8,487 DMPs were identified as hypomethylated. Of these 3,373 (approximately 40%) were significantly hypomethylated, exceeding three standard deviations (3SD) from the healthy control group. No DMPs demonstrated a gain of methylation exceeding 3SD.

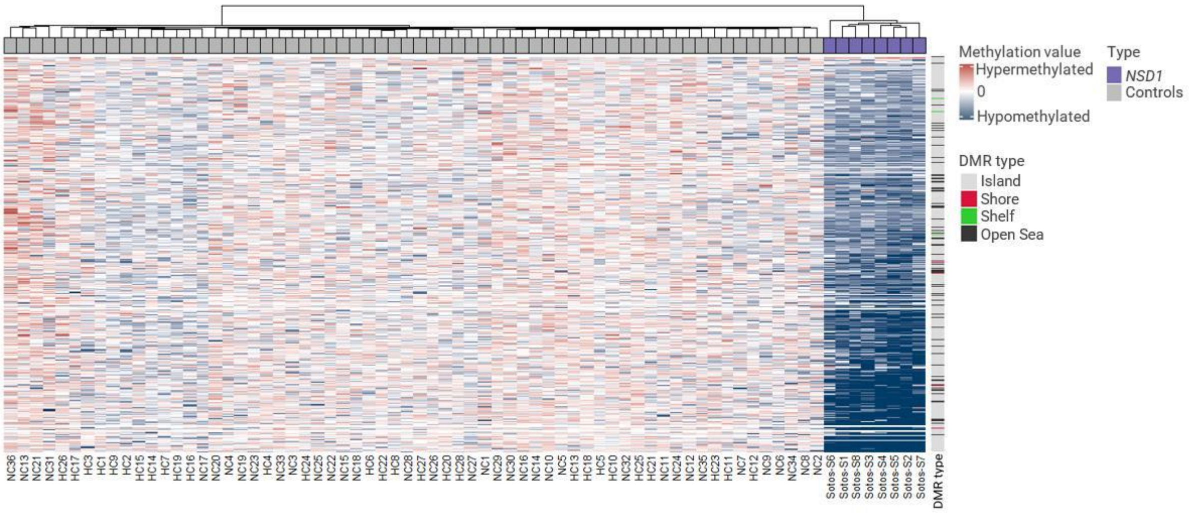

Supplementary Figure 2: Methylation episignature for the familial patient group with *NSD1* exon 3 deletions (n=3). A total of 1,537 significant DMPs were identified control group. Patient 1 showed 551 out of 1,537 DMPs as hypomethylated, while Patients 2 and 3 exhibited an average of 251 out of 1,537 hypomethylated DMPs. No DMPs exhibited a gain of methylation exceeding the 3SD compared to the control group.

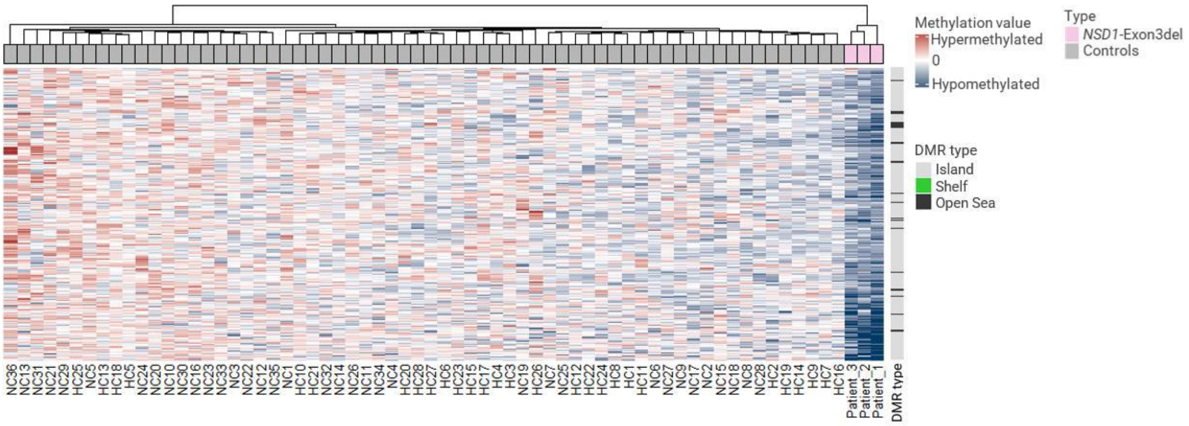

Supplementary Figure 3

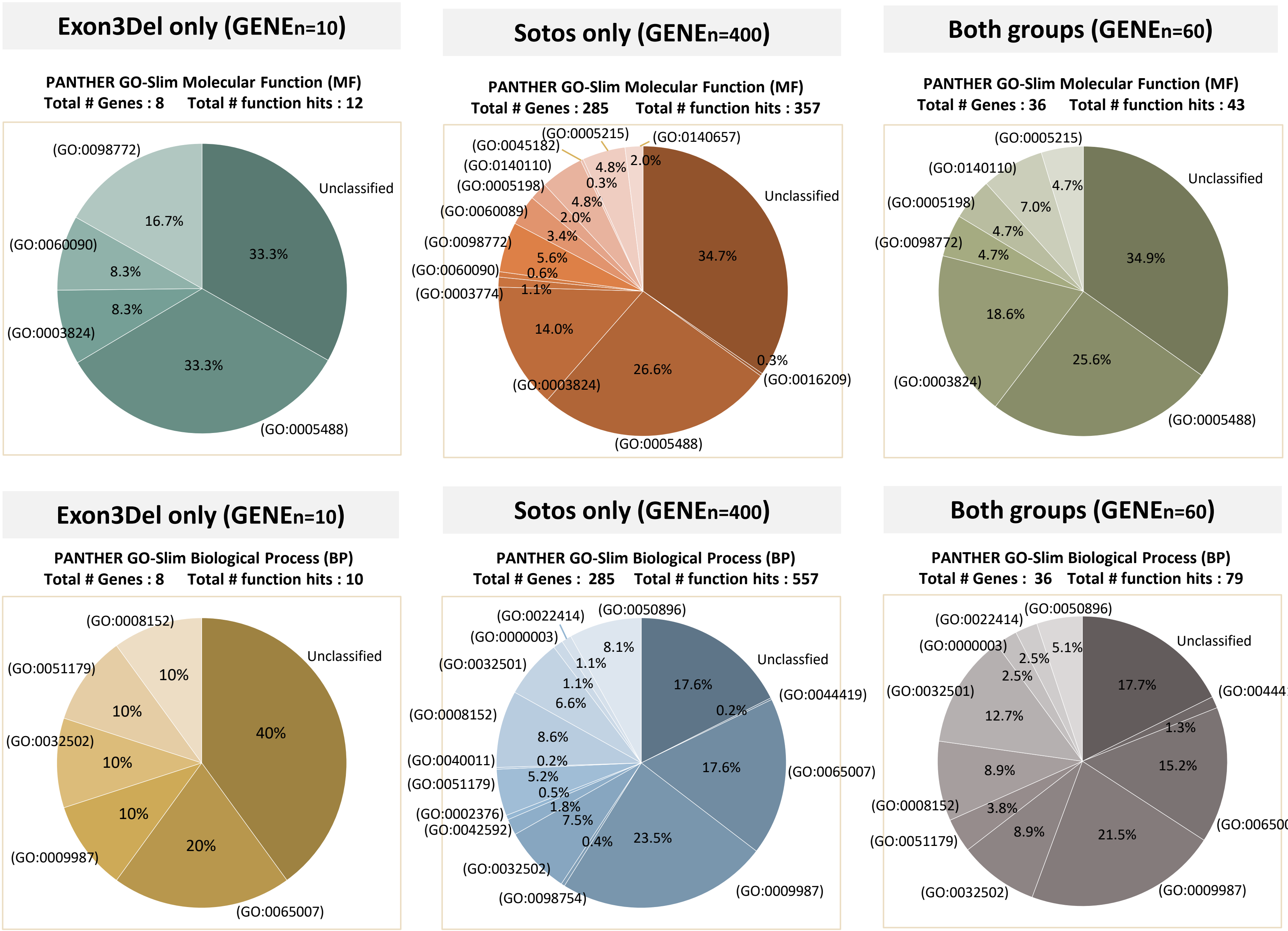

GO:0140657 ATP-dependent activity
GO:0016209 antioxidant activity
GO:0005488 binding
GO:0003824 catalytic activity
GO:0003774 cytoskeletal motor activity
GO:0060090 molecular adaptor activity
GO:0098772 molecular function regulator activity
GO:0060089 molecular transducer activity
GO:0005198 structural molecule activity
GO:0140110 transcription regulator activity
GO:0045182 translation regulator activity
GO:0005215 transporter activity
GO:0044419 biological process involved in interspecies interaction between organisms
GO:0065007 biological regulation
GO:0009987 cellular process
GO:0098754 detoxification
GO:0032502 developmental process
GO:0042592 homeostatic process
GO:0002376 immune system process
GO:0051179 localization
GO:0040011 locomotion
GO:0008152 metabolic process
GO:0032501 multicellular organismal process
GO:0000003 reproduction
GO:0022414 reproductive process
GO:0050896 response to stimulus

Supplementary Figure 3: Gene Ontology (GO) term analysis, after excluding unmapped IDs, for the three groups of genes (those altered in Exon 3 deletion cohort, Sotos only cohort, and both groups)

Supplementary Figure S4

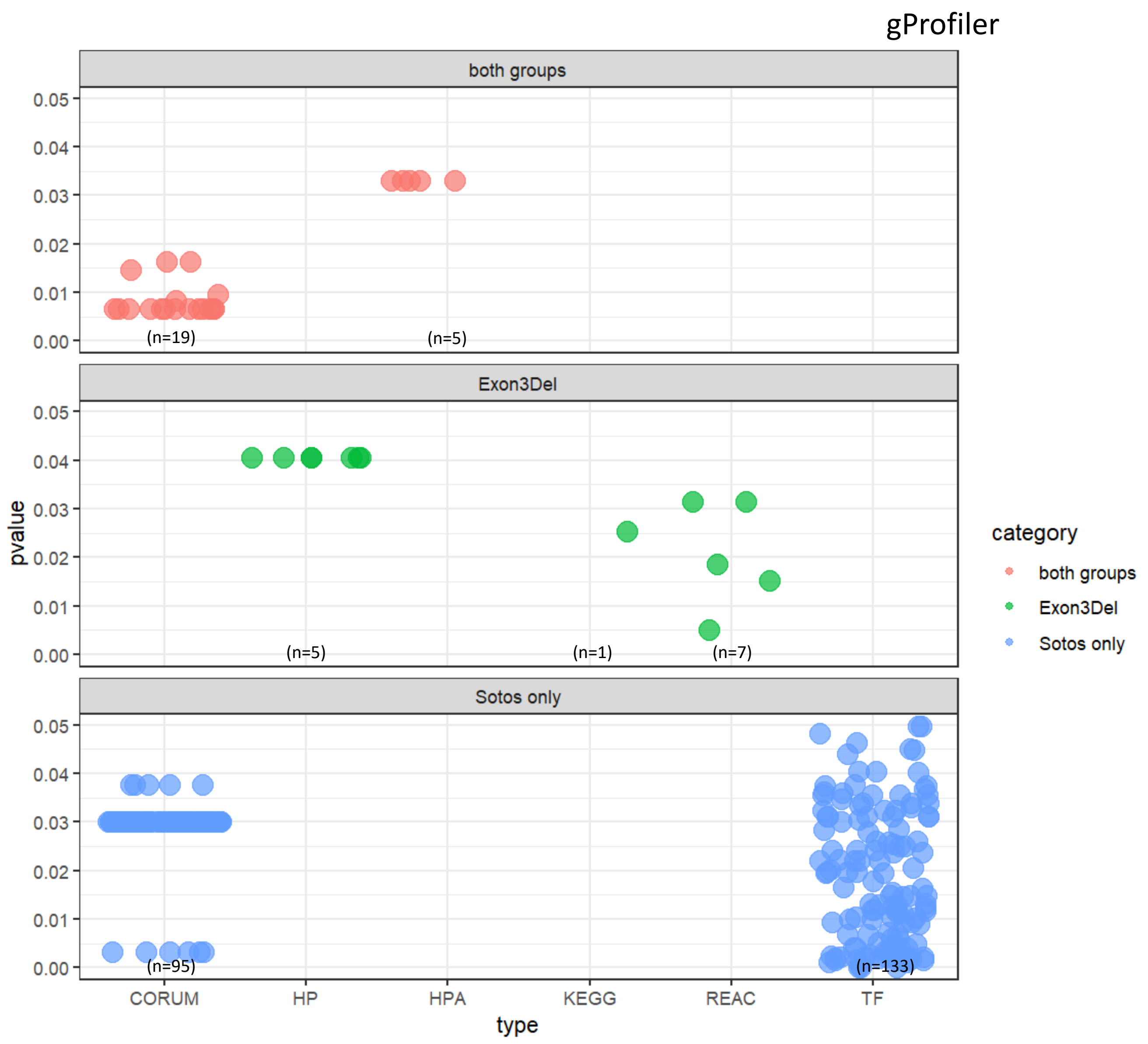

Supplementary Figure 4: Detailed functional profiling by g:Profiler including significant terms and adjusted p-values

Supplementary Tables

Supplementary Table 1: Significant differentially methylated positions (DMPs) (Sotos syndrome patients n=8)

See Excel file Supplementary Table 1

Supplementary Table 2: Overlapping differentially methylated positions (DMPs) between Sotos syndrome patients (n=8) and deletion carriers (n=3)

See Excel file Supplementary Table 2

Supplementary Table 3: Pathway analysis of DMB-associated genes with G2P-linked disease annotations

See Excel file Supplementary Table 3

Supplementary Table 4: Detailed functional profiling by g:Profiler including significant terms and adjusted p-values

See Excel file Supplementary Table 4

Supplementary Table S5: Genes for inherited developmental disorders associated with regions (DMBs) showing significantly altered methylation in individuals with a familial exon 3 deletion (3 individuals; Exon3delonly), classical Sotos syndrome (n=8 individuals; Sotos only) and in both groups (n=11; Both groups). Information on developmental disorder genes was accessed at the Gene2Phenotype resource (https://www.ebi.ac.uk/gene2phenotype/) (accessed 3rd September 2025). HPO (Human Phenotype Ontology) terms (when present in Gene2Phenotype) are recorded for each condition those HPO terms highlighted in red are recorded for both Sotos syndrome and the relevant condition. Relevant HPO terms for frequent features of Sotos syndrome include: tall stature (HP:0000098), macrocephaly (HP:0000256), developmental delay (HP:0001263), Frontal bossing (HP:0002007), Downslanted palpebral fissures (HP:0000494), joint laxity (HP:0001388), scoliosis (HP:0002650), neonatal hypotonia (HP:0001319)

See Excel file Supplementary Table 5
